# Air contamination with A.baumannii during wound dressing change in burn patients but not in surgical patients

**DOI:** 10.1186/2047-2994-4-S1-P62

**Published:** 2015-06-16

**Authors:** S Messler, M Malecki, PC Fuchs, F Mattner

**Affiliations:** 1 Institut für Hygiene, Cologne, Germany; 2 Klinik für Plastische und Ästhetische Chirurgie, Handchirurgie, Schwerbrandverletztenzentrum, Kliniken der Stadt Köln, Cologne, Germany

## Introduction

*A.baumannii* (AB) colonisation and infection is frequent in burn patients and multiple outbreaks especially with multidrug resistant strains are described in burn units as well as in other intensive care units. The understanding of the transmission routes of AB in health care settings is eminent to control outbreaks and prevent transmission. Burn patients are suspected to spread high amounts of pathogens.

## Objectives

Here, we aim to analyze the contamination of the air during wound dressing change in burn patients in comparison to surgical ones.

## Methods

We obtained microbial air samples during wound dressing change in a distance of 1-2 m from the patient in five burn patients. Air samples in rooms of four surgical patients were taken during wound dressing change and/or suction. All patients were known to be colonized with AB. The MAS 100^®^ air sampler was used with blood agar petri dishes. In each case a volume of 500ml was sampled. Isolates were compared with patient isolates via subtyping in two cases.

## Results

Burn patients had between 10 and 59% of their body surface area burned.

During their overall wound dressing changes AB was detected in air samples <100 KBE/m^3^. In the two cases where typing was performed, a clonal relationship to patient isolates was shown. During one dressing change an additional air sample was taken on the floor outside the patient room with no growth of AB. All surgical patients presented only small sutured wounds. No positive air sample was observed during dressing change in these patients. In only two of these patients AB was detected in the patients wound, all were positive in tracheal secretion.

## Conclusion

During wound dressing changes in burn patients colonized with AB, the pathogen was present in the patient surrounding air in low numbers, but not outside the room. This implicates prevention measures like surface cleaning after procedure, wearing of appropriate personal protective equipment, closing doors and potentially air disinfection methods or quarantine for a period of time.

In contrast, patients with closed wounds seem not to disperse any AB during dressing changes, suggesting that the magnitude of an open wound could be predictive for air spreading during agitation via dressing changes rather than colonisation with AB itself.

## Disclosure of interest

None declared.

